# Regulation of proteostasis and innate immunity via mitochondria-nuclear communication

**DOI:** 10.1083/jcb.202310005

**Published:** 2024-02-09

**Authors:** Sookyung Kim, Theresa R. Ramalho, Cole M. Haynes

**Affiliations:** 1 Department of Molecular, Cell and Cancer Biology, University of Massachusetts Chan Medical School, Worcester, MA, USA

## Abstract

Mitochondria are perhaps best known as the “powerhouse of the cell” for their role in ATP production required for numerous cellular activities. Mitochondria have emerged as an important signaling organelle. Here, we first focus on signaling pathways mediated by mitochondria-nuclear communication that promote protein homeostasis (proteostasis). We examine the mitochondrial unfolded protein response (UPR^mt^) in *C. elegans*, which is regulated by a transcription factor harboring both a mitochondrial- and nuclear-targeting sequence, the integrated stress response in mammals, as well as the regulation of chromatin by mitochondrial metabolites. In the second section, we explore the role of mitochondria-to-nuclear communication in the regulation of innate immunity and inflammation. Perhaps related to their prokaryotic origin, mitochondria harbor molecules also found in viruses and bacteria. If these molecules accumulate in the cytosol, they elicit the same innate immune responses as viral or bacterial infection.

## Teaser

Kim et al. review how mitochondria-to-nuclear communication promotes proteostasis and immunity.

## Introduction

As a remnant of an engulfed α-proteobacteria, mitochondria have evolved a dynamic and intertwined relationship with the nucleus that includes multiple signaling pathways that mediate mitochondrial biogenesis and proteostasis as well as innate immunity. Enclosed by two membranes, mitochondria retain a remnant of the prokaryotic genome (mtDNA) that encodes 13 oxidative phosphorylation (OXPHOS) proteins along with the tRNAs and ribosomal RNAs required to synthesize the OXPHOS proteins on mitochondrial ribosomes within the mitochondrial matrix. The genes that encode the additional ∼1,200 mitochondrial proteins are located in the nuclear genome. Following synthesis on cytosolic ribosomes, the proteins are targeted to mitochondria via targeting sequences and imported by well-characterized import complexes (Busch et al., 2023).

Mitochondria are perhaps best known as the powerhouse of the cell as they generate energy in the form of ATP via the respiratory chain complexes and the ATP synthase. Mitochondria are also the site of amino acid, nucleotide, and iron–sulfur cluster synthesis. Here, we review the molecular mechanisms and physiologic impact of diverse pathways mediated by mitochondrial-nucleus communication during cell growth, proteotoxic stress, and pathogen infection.

### Regulation of mitochondrial protein homeostasis and biogenesis by mito-nuclear crosstalk

Retrograde signaling as a response to perturbed mitochondrial function was first described in the budding yeast *Saccharomyces cerevisiae* (Parikh et al., 1987). Deleterious mtDNA mutations or depletion of mtDNAs altered nuclear gene expression resulting in glutamate biogenesis. The changes in gene expression allow mitochondria to generate α-ketoglutarate by supplying acetyl-CoA and citrate synthesized by peroxisomes to mitochondria through anaplerotic reactions. By doing so, retrograde signaling allows mitochondria to continue to supply glutamate when respiration is impaired (Epstein et al., 2001; Butow and Avadhani, 2004). The retrograde response was the first mitochondrial-nuclear communication to be identified leading to the identification of numerous conceptually similar pathways that mediate diverse aspects of biology.

#### The mitochondrial unfolded protein response (UPR^mt^)

Findings in mammalian cells suggested the existence of a mitochondrial-nuclear communication known as the UPR^mt^. Transcription of the nuclear genes that encode the matrix-localized-chaperones Hsp60 and Hsp10 are induced in response to impaired mtDNA replication caused by ethidium bromide exposure (Martinus et al., 1996; Zhao et al., 2002). This observation is conceptually similar to a signaling pathway that mediates the increased transcription of genes encoding endoplasmic reticulum (ER)-localized chaperones in response to proteostasis perturbations within the ER known as the UPR (Walter and Ron, 2011). Thus, the mitochondrial-specific response was coined the UPR^mt^ (Benedetti et al., 2006).

While first identified in mammalian cells, the regulation of mitochondrial-to-nuclear communication was initially elucidated using *C. elegans*. Mitochondrial-nuclear communication pathways exist in both model systems and are regulated by conceptually similar processes. However, the diversity of responses and forms of regulation are expanded in mammals. This surveillance system promotes mitochondria maintenance and function by promoting proteostasis via the transcription of mitochondrial chaperones, proteases, and components of the mitochondrial protein import complexes required for chaperone import and mitochondrial biogenesis. Here, we describe both the similarities as well as differences between signaling in *C. elegans* and mammals.

**Figure 1.**
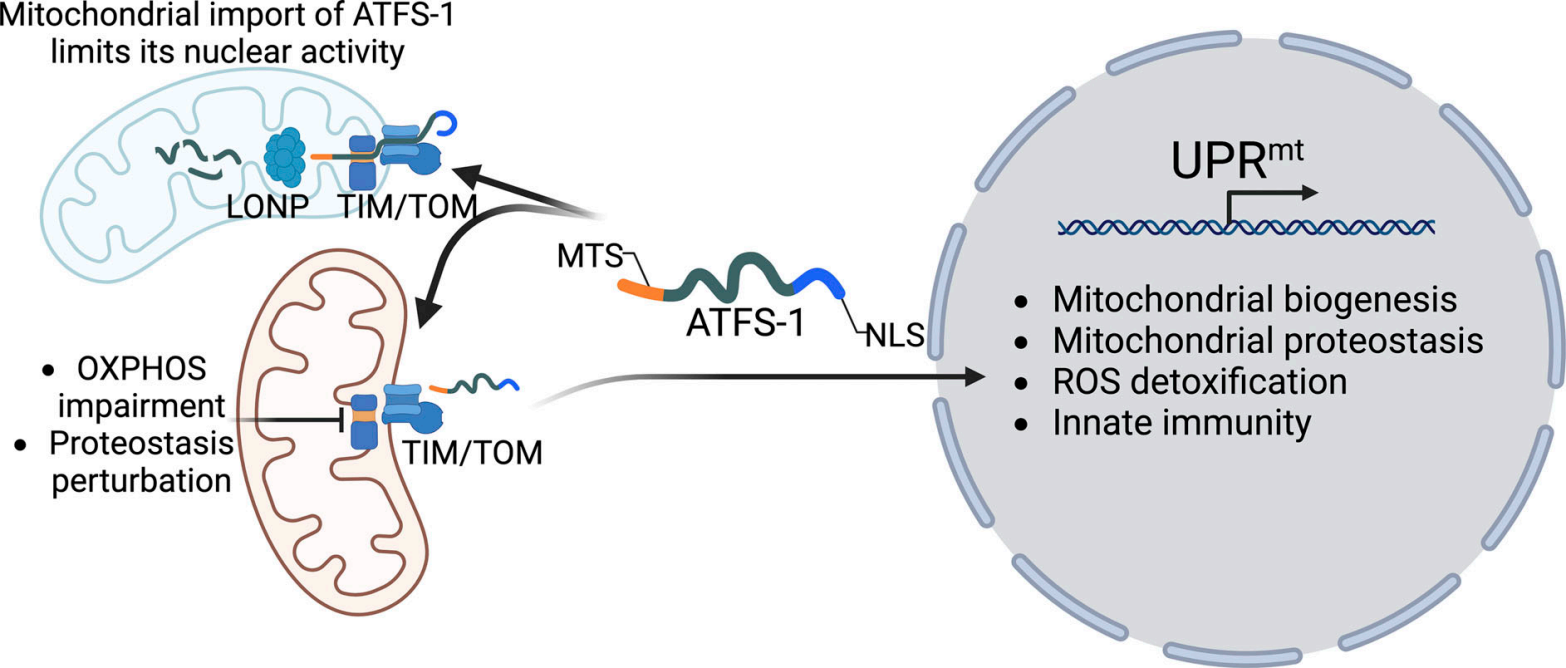
**Regulation of the mitochondrial unfolded protein response (UPR**^**mt**^**) in *C. elegans*.** The bZIP transcription factor ATFS-1 harbors an amino-terminal MTS and a nuclear localization sequence (NLS) within the bZIP domain near the C-terminus. During cell growth, the majority of ATFS-1 is imported into the mitochondrial matrix via the TIM and TOM translocases and subsequently degraded by the protease LONP. However, if mitochondrial protein import capacity is reduced due to mitochondrial dysfunction or high levels of OXPHOS protein import, ATFS-1 import is impaired, causing it to accumulate in the cytosol and traffic to the nucleus, where it regulates the transcription of over 600 genes that promote mitochondrial proteostasis and biogenesis, glycolysis, ROS detoxication, and innate immunity.

In *C. elegans*, UPR^mt^ is mediated by numerous proteins including the basic leucine zipper (bZip) transcription factor ATFS-1, which was discovered via an RNAi screen (Haynes et al., 2010). Importantly, ATFS-1 harbors both an amino-terminal mitochondrial targeting sequence (MTS) as well as a nuclear localization sequence (NLS) located near the C-terminus. During cell growth and development, the majority of ATFS-1 is imported into the matrix via the Translocase of the Outer Membrane (TOM) and Translocase of the Inner Membrane (TIM) complex, similar to proteins comprising the OXPHOS complexes and TCA cycle. Upon entering the matrix, the MTS is cleaved from ATFS-1, which is followed by degradation via the matrix-localized protease LONP (Nargund et al., 2015) (Fig. 1).

Protein import into mitochondria as well as protein degradation by LONP requires ATP. Thus, both the import of ATFS-1 into mitochondria and the degradation of ATFS-1 within the matrix require a functional OXPHOS system. Conditions that perturb mitochondrial proteostasis or OXPHOS function limit the amount of ATFS-1 imported into mitochondria. As a result, a fraction of ATFS-1 accumulates in the cytosol and is instead trafficked to the nucleus via the NLS where it regulates the transcription of over 600 genes (Fig. 1). Presumably, this activity allows cells to evaluate the function of the entire mitochondria network. If the mitochondrial import capacity in each cell is able to efficiently import and degrade ATFS-1, the mitochondrial network is perceived to be functional to meet cellular metabolic requirements. Intriguingly, ATFS-1 harbors a relatively weak MTS relative to OXPHOS or chaperone proteins potentially allowing UPR^mt^ activation prior to the complete degeneration of the mitochondrial network (Shpilka et al., 2021; Rolland et al., 2019). Following trafficking to the nucleus, ATFS-1 binds promoters harboring UPR^mt^ elements leading to transcription of a program that, in addition to proteostasis components, includes over 400 genes required for mitochondrial network recovery and/or biogenesis (Nargund et al., 2015). Presumably, the UPR^mt^ activation is reduced as the mitochondrial import capacity recovers and ATFS-1 can be imported and degraded.

In addition to ATFS-1, several additional transcription factors mediate mito-nuclear communication. Interestingly, heat shock factor 1 (HSF1), which is best characterized as regulating the expression of cytosol-localized molecular chaperones in response to heat or conditions that increase unfolded or misfolded protein accumulation within the cytosol, is also activated in response to mitochondrial perturbations (Sutandy et al., 2023). HSF1-dependent transcription is impaired via interactions with the cytosolic chaperone HSP70 and its co-chaperone DNAJA1. HSF1 is activated in response to the combination mitochondria-generated reactive oxygen species (ROS) and the accumulation of unfolded mitochondrial precursor protein in the cytosol due to reduced mitochondrial import capacity. Cytosolic ROS accumulation oxidizes cysteine residues within DNAJA1, causing HSP70 to release HSF1 and allowing it to traffic to the nucleus and activate transcription of several genes that encode mitochondrial chaperones. These findings establish a ROS-mediated mito-nuclear communication pathway that is conceptually similar to findings in *C. elegans*, demonstrating that HSF1 is also activated in response to mitochondrial stressors that increase cytosolic ROS but may also have been caused by the accumulation of mitochondrial precursor proteins in the cytosol (Kim et al., 2016a). Moreover, the transcription cofactor GPS2 has also been shown to regulate mito-nuclear communication. In response to the depolarization of the mitochondrial inner membrane, GPS2 is desumoylated by SENP1, allowing GPS2 to traffic to the nucleus where it induces transcription of genes required for mitochondrial biogenesis (Cardamone et al., 2018). Lastly, the ubiquinone biosynthesis protein CLK-1 has been suggested to function as a rheostat to modulate both mitochondrial ROS metabolism and the UPR^mt^ in response to cellular ROS; however, the mechanism of regulation remains unclear (Monaghan et al., 2015).

#### The Integrated Stress Response (ISR) and mito-nuclear communication

The ISR is a translation control pathway mediated by four protein kinases. In response to diverse cellular stressors including starvation, ER stress, and viral infection, the activated kinase phosphorylates the translation initiation factor eIF2α, resulting in a reduction in the rate of general protein synthesis and preferential translation of mRNAs harboring upstream open reading frames, or uORFs, such as the transcription factors ATF4, CHOP, and ATF5. Each of the ISR kinases (GCN2, PERK, PKR, and HRI) has been shown to be activated during mitochondrial dysfunction. The roles of the ER stress-sensitive kinase PERK and GCN2, which is stimulated by amino acid depletion or ribosome stalling, have been reviewed elsewhere (Baker et al., 2012; Monteiro et al., 2023). Here, we focus on HRI and PKR, both of which are directly induced via mitochondrial perturbations.

**Figure 2.**
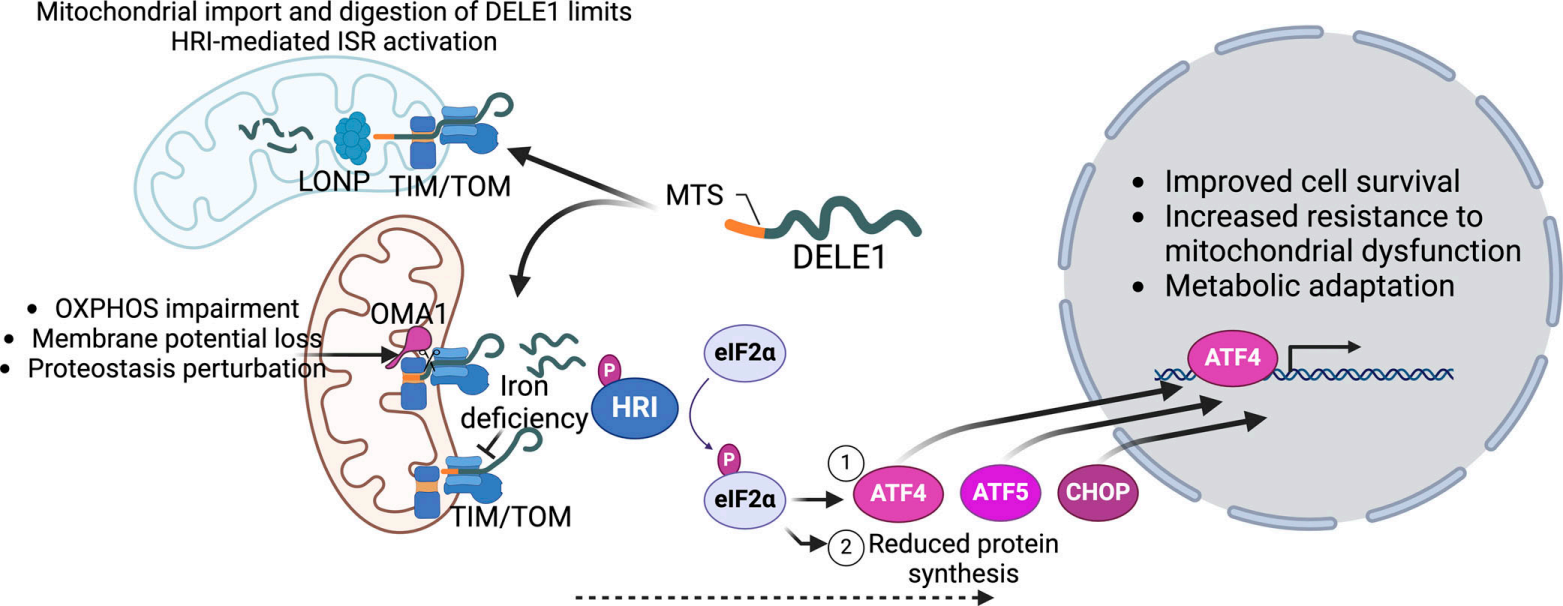
**Regulation of the ISR during mitochondrial stress.** The protein DELE1 harbors an amino-terminal MTS and is constitutively imported into functional mitochondria where it is degraded by the protease LONP. However, during mitochondrial stress caused by OXPHOS perturbations or depletion of the mitochondrial inner membrane potential, DELE1 import is stalled with the C-terminal domain remaining in the cytosol. Iron deficiency causes DELE1 to remain in the TOM channel, while inner membrane uncoupling causes OMA1 to cleave DELE1 allowing the C-terminal fragment to diffuse back into the cytosol. Cytosolic DELE1 oligomerizes, binds, and activates the ISR kinase HRI. Subsequent phosphorylation of eIF2α (1) reduces the rate of total protein synthesis while (2) increasing the translation of three transcription factors (ATF4, CHOP, and ATF5) that promote survival during mitochondrial dysfunction.

HRI was originally discovered for its role in red blood cell development and maturation as it coordinates globin protein synthesis with heme levels (Das et al., 1979). More recently, a direct link between mitochondrial function and HRI activation has been identified. Independent mutagenesis screens identified HRI, the inner mitochondrial membrane-localized protease OMA1, and the previously unstudied protein DELE1 as being required for ATF4 or CHOP translation during mitochondrial perturbation caused by inner membrane depolarization by CCCP, which impairs mitochondrial protein import (Fessler et al., 2020; Guo et al., 2020). Intriguingly, DELE1 was found to harbor an amino-terminal MTS and has a relatively short-half life as it is degraded within the mitochondrial matrix by LONP1 (Sekine et al., 2023), reminiscent of the mechanism by which ATFS-1 is negatively regulated (Fessler et al. 2022). However, upon inner membrane depolarization, DELE1 fails to cross the mitochondrial inner membrane and is cleaved by OMA1 within the intermembrane space. In turn, the C-terminal fragment DELE1 enters the cytosol where it oligomerizes (Yang et al., 2023) and directly binds HRI, stimulating eIF2α phosphorylation and ISR activation (Fig. 2).

A number of stressors that activate the DELE1-mediated ISR have been identified. For example, activation of HRI during iron deficiency requires DELE1 (Sekine et al., 2023). Upon iron depletion, DELE1 accumulates on the mitochondrial outer membrane where it activates HRI (Fig. 2). Recent evidence indicates that a domain within DELE1 (amino acids 102–200) is required for impaired import of DELE1 upon iron depletion. Importantly, the DELE1-HRI pathway protects against cell death caused by iron depletion (Sekine et al., 2023).

Beyond reducing the rate of protein synthesis during mitochondrial stress, the transcriptional outputs that promote survival remain largely unclear. However, DELE1-dependent ISR activation promotes ATF4 translation, which activates the transcription of genes that mediate one-carbon metabolism (Bao et al., 2016; Ducker and Rabinowitz, 2017; Huynh et al., 2023), to protect against mitochondrial stress. DELE1 was also found to be protective in a mouse model of mitochondrial myopathy (Huynh et al., 2023) and also prevented cell death by ferroptosis caused by impaired expression of the complex IV assembly factor Cox10 (Ahola et al., 2022). Furthermore, DELE1 is required for ISR activation upon mtDNA cleavage or fragmentation, which also involves HRI. Interestingly, the recovery of mtDNA content occurs independently of ATF4 (Fu et al., 2023). These findings suggest that the reduced rate of general protein synthesis or the increased translation of mRNAs that harbor uORFs plays a role in the maintenance or recovery of mitochondrial function in response to mtDNA damage.

In addition to ATF4, CHOP and ATF5 are both translated upon eIF2α phosphorylation due to the presence of uORFs in the 5′ untranslated region of each transcript. Interestingly, like ATFS-1 in *C. elegans*, ATF5 also harbors a relatively weak MTS at the amino terminus, suggesting that it may be further regulated by mitochondrial stress following synthesis. Consistent with this observation, overexpressed ATF5 localizes to mitochondria and nuclei. Work from our group found that ATF5 can rescue UPR^mt^ activation in *C. elegans* lacking ATFS-1, suggesting that both transcription factors are regulated similarly following translation (Fiorese et al., 2016). Furthermore, reduced expression of ATF5 impaired respiration and reduced levels of mtDNAs in mammalian cells. Intriguingly, HSF1 regulates transcription of *atf5* during mitochondrial perturbation, suggesting multiple levels of UPR^mt^ regulation in mammals (Sutandy et al., 2023).

#### Chromatin remodeling mediated by mito-nuclear communication

During *C. elegans* development, chromatin is organized to promote the transcription of genes required for cell differentiation and growth (Mutlu et al., 2018). Cells maintain and adjust mitochondria according to cell development, identity, and changing metabolic needs. However, during early adulthood, chromatin is remodeled to limit transcription in somatic cells to shift resources to germline proliferation and embryo formation. For example, UPRmt is no longer inducible in adulthood as compared with the developmental state when mitochondrial perturbation can activate UPRmt. Intriguingly, when exposed to mitochondrial perturbations during development, chromatin status remains in the developmental state into adulthood, permitting transcription in somatic cells which promotes lifespan extension (Tian et al., 2016; Merkwirth et al., 2016). For example, mitochondrial perturbations such as OXPHOS complex IV inhibition via *cco-1*(RNAi) promote chromatin rearrangements that allow *atfs-1*-dependent transcription and UPR^mt^ activation (Dillin et al., 2002).

During mitochondrial stress, the cytosolic protein LIN-65 translocates to the nucleus in a manner requiring the cytosol-localized histone methyltransferase MET-2 (Tian et al., 2016). In the nucleus, LIN-65 and MET-2 promote methylation of histone H3K9, resulting in global gene-silencing via chromatin compaction (Tian et al., 2016) while the sites where homeobox protein DVE-1 binds remain open, allowing transcription of mitochondrial stress response genes (Haynes et al., 2007) via ATFS-1 (Tian et al., 2016). Importantly, DNA binding by DVE-1 is further regulated by SUMOylation or ubiquitin-like protein UBL-5 binding. UBL-5 binding to DVE-1 promotes transcription (Benedetti et al., 2006), while SUMOylation of DVE-1 antagonizes transcription (Gao et al., 2019). SUMOylation is regulated by the cytosol-localized SUMO-peptidase ULP-4, which deSUMOylates DVE-1 and ATFS-1 (Gao et al., 2019). Interestingly, nuclear localization of LIN-65 requires the mitochondrial matrix-localized quality control protease CLPP-1 (Haynes et al., 2007), consistent with chromatin status being regulated via mitochondrial-nuclear crosstalk (Tian et al., 2016; Mutlu et al., 2018). In addition, UPR^mt^ activation also requires the Jumonji domain histone lysine demethylases, JMJD1.2 and JMJD3.1 in *C. elegans*, or PHF8 and JMJD3 in mammals, with overexpression of either being sufficient to activate the UPR^mt^ (Merkwirth et al., 2016). The transcriptional coactivator CBP/p300 functions downstream of the histone demethylases to promote ATFS-1-dependent transcription (Li et al., 2021).

Numerous mitochondrial metabolites, such as acetyl-CoA, NAD+, and s-adenosyl-methionine are required to generate epigenetic marks that regulate chromatin dynamics and gene expression (Ryall et al., 2015; Wang et al., 2017; Mentch et al., 2015). For example, the TCA cycle substrate acetyl-CoA provides the acetyl groups required for histone acetylation (Shi and Tu, 2015). Furthermore, an increase in the ratio of acetyl-CoA to CoA promotes the activity of histone acetyltransferases and histone acetylation (Montgomery et al., 2016). Intriguingly, during mitochondrial stress, acetyl-CoA levels decrease due to reduced TCA cycle activity, which induces the histone deacetylase complex (NuRD) and DVE-1 expression, promoting UPR^mt^ activation (Zhu et al., 2020). Furthermore, increased availability of acetyl-CoA prevents longevity caused by mitochondrial perturbations in *C. elegans* potentially by impairing UPR^mt^ activation. Combined, these findings highlight a link between chromatin regulation, metabolite levels, and cellular metabolism (Lee et al., 2014; Moussaieff et al., 2015; Jo et al., 2020).

#### Intercellular signaling via mitokines regulates mito-nuclear crosstalk

In addition to cell-autonomous signaling pathways, intercellular or inter-tissue communication also regulates mitochondrial function by promoting mito-nuclear communication, which is regulated by secreted molecules known as mitokines (Berendzen et al., 2016; Chen et al., 2021; Zhang et al., 2018; Shao et al., 2016). Mitokines transmit signals from cells with stressed mitochondria to otherwise non-stressed neighboring or distal cells and induce UPR^mt^ in a cell non-autonomous manner. Pioneering studies in *C. elegans* demonstrated that mitochondrial perturbations caused by polyglutamine expression within neurons resulted in mitochondrial stress and UPR^mt^ activation in neurons as well as in intestinal cells, which promotes lifespan extension (Durieux et al., 2011) (reviewed here [Zhang et al., 2023a]). In addition, mitokine signaling from neurons can induce UPR^mt^ and increase mtDNA replication (Zhang et al., 2021) in the germline (Calculli et al., 2021). UPR^mt^ regulation via intercellular communication has been thoroughly reviewed (Bar-Ziv et al., 2020).

### Mitochondria-nuclear crosstalk and innate immunity

In addition to regulating mitochondrial proteostasis and biogenesis, mitochondria-nuclear communication regulates both the initiation and downregulation of multiple innate immunity signaling pathways. For example, findings in *C. elegans* demonstrated that mitochondrial dysfunction caused by inhibition of oxidative phosphorylation caused by human pathogens such as *Pseudomonas aeruginosa* induced UPR^mt^-dependent expression of antibacterial genes (Pellegrino et al., 2014; Jeong et al., 2017), suggesting roles for mitochondria in regulating innate immunity (Melo and Ruvkun 2012; Liu et al., 2014).

Due to their bacterial origin, mitochondria harbor numerous molecules also found in diverse bacterial species (Krysko et al., 2011). In general, innate immune signaling is initiated when a DAMP (damage-associated molecular pattern) or viral/bacterial PAMP (pathogen-associated molecular pattern) binds a pattern recognition receptor (PRR) in the cytosol or the cellular membrane (Lyu et al., 2023). Self-recognition of mitochondrial DAMPs in the cytosol results in innate immune pathway activation. For example, the inner mitochondrial membrane lipid cardiolipin, which is also present in both gram-negative and gram-positive bacteria (Oemer et al., 2018), activates innate immune pathways (Elliott et al., 2018) if it accumulates within the outer mitochondrial membrane (Elliott et al., 2018). Furthermore, transcription of circular mtDNAs occurs simultaneously on each strand, allowing for the accumulation of double-stranded RNAs (Bogenhagen and Yoza, 1986). During mitochondrial stress, mtDNA and/or mtDNA-derived double-stranded RNAs (mtRNA) can accumulate in the cytosol, triggering nucleic acid sensors and the activation of innate immunity pathways (Sun et al., 2013, 2017; West et al., 2015; Lei et al., 2021, 2023; Tigano et al., 2021; Torres-Odio et al., 2021). Thus, mitochondrial maintenance and efficient mitochondrial biogenesis via the pathways described in the previous section are essential to retain DAMPs within mitochondria to limit immune response activation in the absence of pathogens.

#### Accumulation of mtDNAs in the cytosol activates cGAS-STING

**Figure 3.**
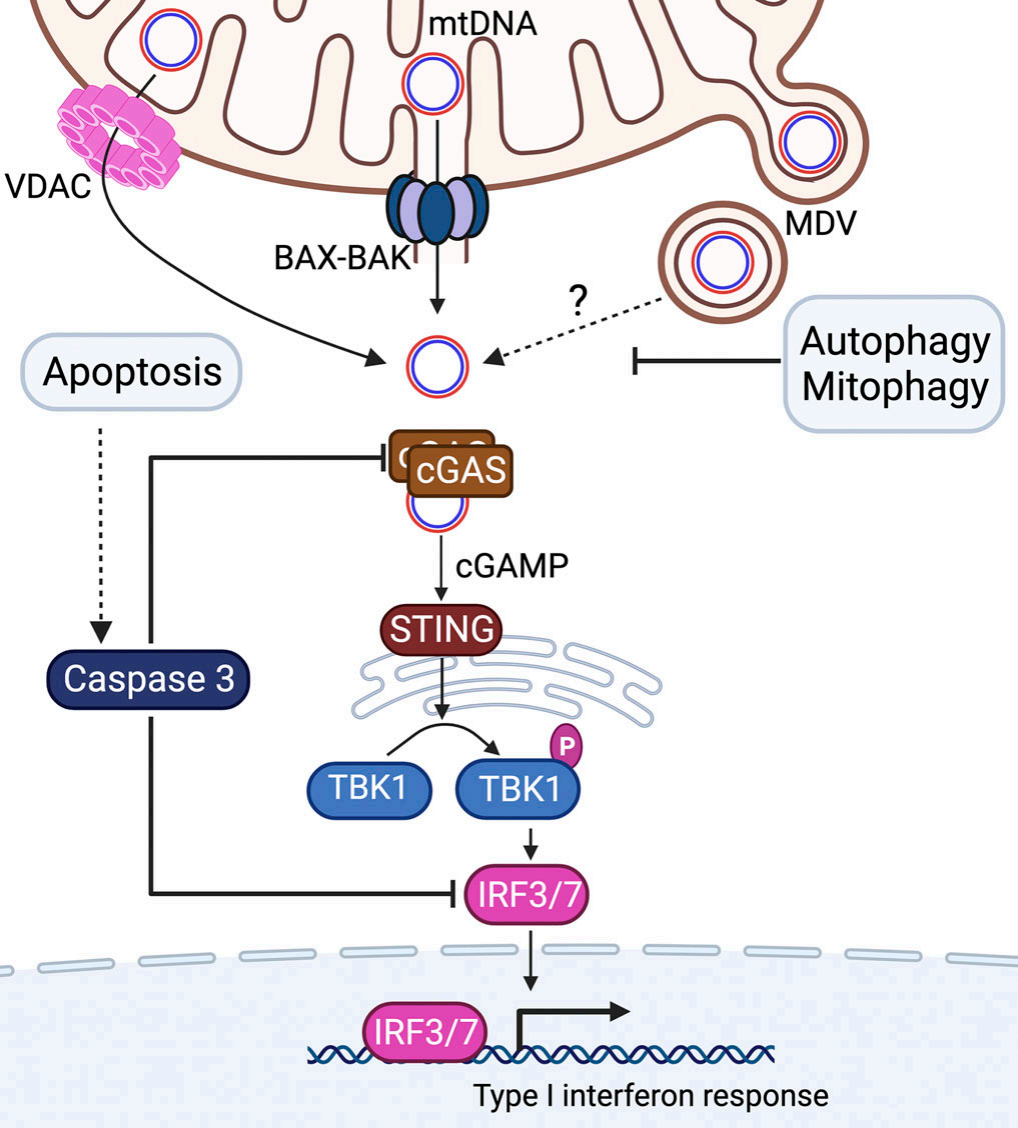
**Accumulation of mtDNAs in the cytosol stimulates the cGAS-STING-mediated immune response.** mtDNAs reside in the mitochondrial matrix. However, mtDNAs enter the cytosol upon pore formation in the mitochondrial outer membrane by VDAC oligomerization or BAX–BAK assembly. Mitochondrial-derived vesicles (MDVs) can form during mitochondrial stress, providing a third route by which mtDNAs exit mitochondria. In the cytosol, mtDNAs stimulate cGAS, which generates cGAMP to activate STING, which phosphorylates TBK1. In turn, the transcription factors IRF3 and IRF7 induce the type I interferon immune response. Importantly, apoptosis along with caspase 3 antagonizes the interferon response by cleaving cGAS, IRF3, and IRF7. Mitophagy also antagonizes the response by degrading damaged mitochondria which limits the accumulation of mtDNAs in the cytosol.

mtDNAs are localized within the matrix and associated with the inner mitochondrial membrane. mtDNAs are bound by the high mobility group protein TFAM that packages the genome by binding to G quadruplex structures located throughout mtDNA (Lyonnais et al., 2017). TFAM is required for both mtDNA replication and transcription (Dairaghi et al., 1995; Rantanen and Larsson, 2000). Intriguingly, TFAM heterozygous mice have aberrant mtDNA packaging, which causes the accumulation of mtDNAs in the cytosol and an increase in antiviral signaling (West et al., 2015). In the cytosol, mtDNAs interact with the double-stranded DNA sensor cGAS (cyclic GMP-AMP synthase) (Sun et al., 2013) (Fig. 3). Interestingly, naked dsDNA and dsDNA with specific curvature mediated by TFAM are more potent activators of cGAS than histone-bound dsDNA from the nucleus (Andreeva et al., 2017; Zierhut et al., 2019). Once synthesized by activated cGAS, cGAMP binds STING (stimulator of interferon genes), which resides on the cytosolic surface of the ER. In turn, STING stimulates the activation of the transcription factors IRF3 and IRF7 (Interferon Regulatory Factor), resulting in increased expression of type I interferon genes. The binding of type I interferons to their cognate receptors induces the transcription of interferon-stimulated genes (ISGs) and senescence-associated secretory phenotype (SASP) (Victorelli et al., 2023). mtDNA accumulation in the cytosol causes SASP expression via cGAS-STING activation and reduces lifespan in mice (Victorelli et al., 2023). Alternatively, the Z-form of mtDNA, which is increased upon TFAM depletion, is stabilized by Z-DNA binding protein 1 (ZBP1). The mtDNA-ZBP1 complex induces ISG expression and cell death by increasing cGAS activity (Lei et al., 2023). Here, ZBP1 is required to activate the inflammatory response caused by chemotherapy, which perturbs mtDNA stability.

Several mechanisms allow mtDNAs to exit mitochondria into the cytosol. During apoptosis, mtDNAs can reach the cytosol via BAX- and BAK-mediated pore formation in the outer mitochondrial membrane, resulting in mitochondrial outer membrane permeabilization (MOMP) (Riley et al., 2018) (Fig. 3). Herniation of the inner mitochondrial membrane through the OMM pores, followed by degeneration of the herniated inner membrane, releases matrix components into the cytosol such as mtDNAs (McArthur et al., 2018) and mtRNAs (Dhir et al., 2018), causing cGAS-STING activation and type I interferon gene expression. Importantly, pore formation in a fraction of mitochondria, known as minority MOMP, results in innate immune activation without cell death (Ichim et al., 2015; Brokatzky et al., 2019; Victorelli et al., 2023) This sublethal MOMP has been demonstrated to stimulate the immune response by increasing mtDNA instability and introducing mitochondrial DAMPs into the cytosol (Ichim et al., 2015; Brokatzky et al., 2019; Victorelli et al., 2023).

Pore formation in the mitochondrial outer membrane can also occur independent of BAX and BAK (Flores-Romero et al., 2022; Kim et al., 2019). In stressed mitochondria, voltage-dependent anion channels (VDAC) oligomerize to form a pore through which mtDNAs can enter the cytosol. Intriguingly, pharmacologic inhibition of VDAC oligomerization also impairs the mtDNA-mediated immune response (Kim et al., 2019). Furthermore, the amino-terminal domain of VDAC has been shown to interact with mtDNA, which promotes VDAC oligomerization. However, it remains unclear how mtDNAs reach the intermembrane space where they can interact with VDAC (Kim et al., 2019), but the mitochondrial permeability transition pore (mPTP) may be involved (Yu et al., 2020; Xian et al., 2022; Zhang et al., 2022).

A third mechanism by which mtDNAs reach the cytosol is via mitochondrial-derived vesicles (MDVs), which are small vesicles originally discovered for their role in mitochondrial protein quality control (Soubannier et al., 2012; König et al., 2021). MDV formation is initiated via the MIRO-dependent generation of mitochondrial outer membrane protrusions along microtubules, which is followed by the recruitment of the GTPase DRP1, membrane scission, and vesicle formation (König et al., 2021). Importantly, cargo found within MDVs include mtDNAs as well as mtRNAs (Caielli et al., 2016). It remains unclear how mtDNAs or mtRNAs exit MDVs to interact with the nucleic acid sensors in the cytosol. Interestingly, fumarate accumulation caused by inhibition of the TCA cycle protein fumarate hydratase causes MDV formation (Zecchini et al., 2023). Exogenous fumarate is sufficient to drive MDV formation (Zecchini et al., 2023). MDV formation allows mtDNA and mtRNA accumulation in the cytosol and activation of the cGAS-STING-dependent and mtRNA-mediated innate immune responses (Fig. 3). While mtDNA depletion mitigated fumarate-dependent induction of the immune response, TFAM levels were unchanged suggesting that the fumarate-dependent immune response is not mediated by TFAM depletion. Consistent with the requirement for MDVs, inhibition of mitochondrial cargo packaging into MDVs impaired the fumarate-dependent immune response (Zecchini et al., 2023; Todkar et al., 2021). Importantly, MDV-driven cGAS-STING activation occurs independently of BAX and BAK. However, the mechanism(s) by which fumarate accumulation engages the machinery to generate MDVs remains unclear.

Intriguingly, the introduction of exogenous fumarate reduces the secretion of proinflammatory cytokines by immune cells (Zinger et al., 2022; Zhang et al., 2023b; Brennan et al., 2015). Furthermore, the production of intracellular fumarate also activates the ISR and promotes mitochondrial network recovery (Quirós et al., 2017). In addition to promoting the release of mtDNAs into the cytosol, fumarate also induces protective pathways to restore the mitochondrial network and limit the synthesis of proinflammatory cytokines. Consistent with these observations, the introduction of exogenous fumarate has been shown to have anti-inflammatory effects and limit oxidative stress in diseases characterized by systemic inflammation (Zinger et al., 2022; Loewe et al., 2002; Stoof et al., 2001; Timpani and Rybalka 2020; McGuire et al., 2016).

#### Inflammasome activation by mitochondrial components

Inflammasomes are a diverse group of protein complexes that assemble in the cytosol in response to a variety of DAMPs and PAMPs to produce proinflammatory cytokines such as IL-1β and IL-18 (Martinon et al., 2002; Lamkanfi and Dixit, 2014).

The inflammasome is comprised of the NOD-like receptor (NLR), adapter proteins, and caspases (Okin and Kagan, 2023). NLRs are PRR sensing molecules characterized by the presence of a nucleotide-binding domain and the NACHT (NAIP, CIITA, HET-E, TEP1) domain, which is required for oligomerization. Upon ligand binding, assembled inflammasomes activate caspases that cleave cytokines to promote their maturation. Alternatively, caspases can also cleave gasdermin D (GSDM D), which forms pores in the plasma membrane to induce a lytic type of cell death known as pyroptosis (Okin and Kagan, 2023; Bauernfeind et al., 2009; He et al., 2015). Importantly, the transcription factor NF-κB is required for the expression of the complex components and immature forms of interleukins (IL)-1β, IL-18, and GSDM D (Bauernfeind et al., 2009).

**Figure 4.**
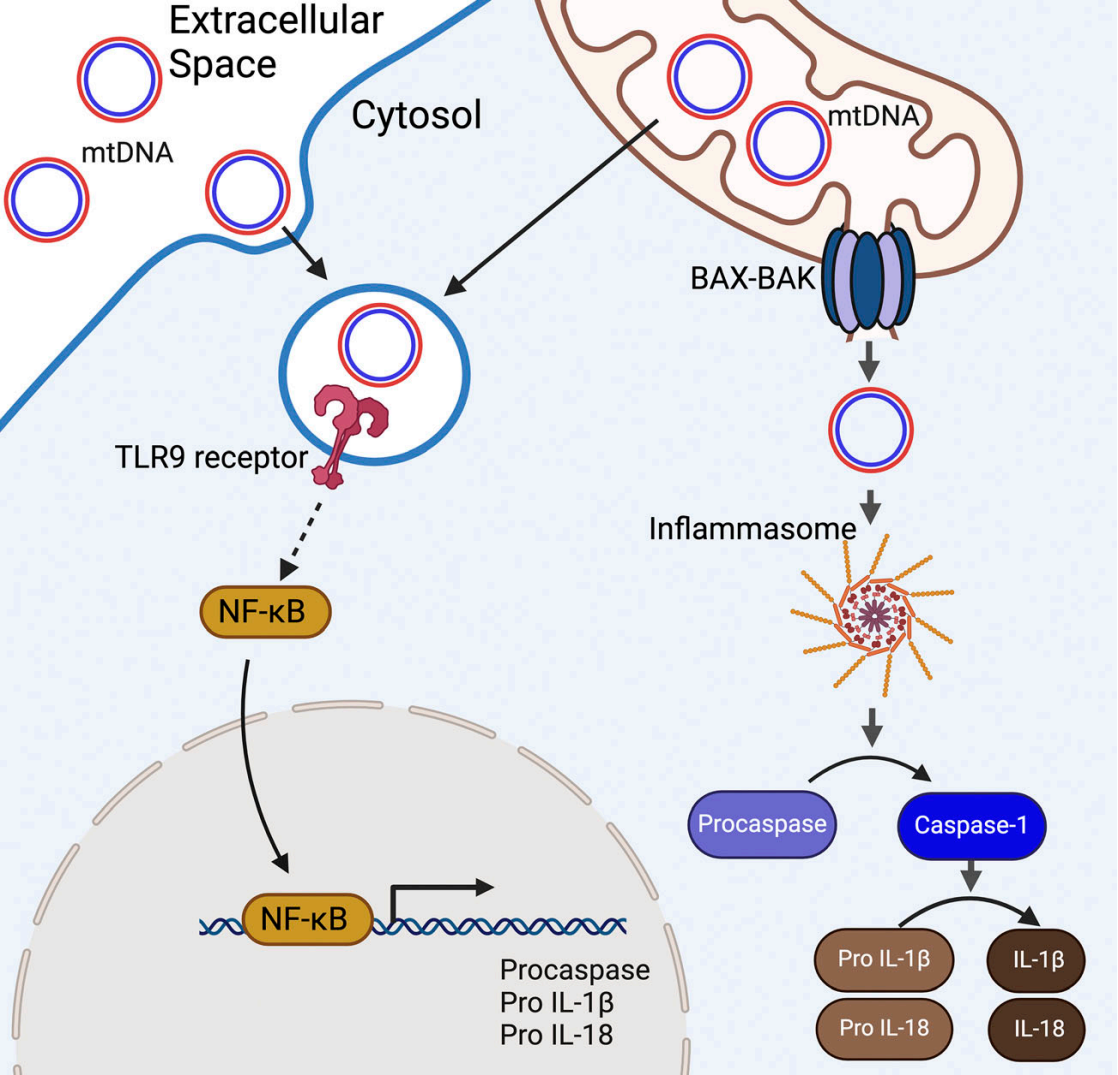
**Cytosolic mtDNAs activate inflammasomes.** Extracellular mtDNAs may be endocytosed by a cell. Alternatively, vesicles that escaped degradation by autophagy or mitochondrial-derived vesicles may carry mtDNAs after budding out from the mitochondria. mtDNA present on the endosome can activate TLR9 receptor. Downstream signaling of activated TLR results in the generation of NF-κB, which primes the cell for inflammasome-mediated immune response by increasing expression of immature cytokines. In parallel, cytosolic mtDNA can be sensed by PRR molecules for inflammasome. Sensing cytosolic mtDNA, two PRRs, NLRP3 and AIM2, have been shown to initiate the assembly of inflammasome. When produced, inflammasome stimulates the maturation of proinflammatory cytokines such as IL-1β and IL-18 by activating caspase-1.

The specificity of an inflammasome is mediated by a pattern-specific ligand that activates different types of NLRs (Takeuchi and Akira, 2010). The NLRs are grouped into four subtypes (NOD, NLRPs, IPAF, and PYHIN) that recognize different ligands. NLRP3 is the best-characterized inflammasome activated by mitochondrial DAMPs such as ROS, mtDNA, or cardiolipin (Muruve et al., 2008; Shimada et al., 2012; Nakahira et al., 2011; Seoane et al., 2020) (Fig. 4). Both oxidized (Shimada et al., 2012) and unoxidized (Zhong et al., 2018) mtDNAs have been reported to activate NLRP3 and PYHIN AIM2-inflammasomes. However, both inflammasomes can also be activated by ROS. Prolonged inflammasome activation further increases the cytosolic accumulation of mtDNA via a feed-forward loop that requires ROS (Nakahira et al., 2011; Zhong et al., 2018). Thus, diverse forms of mitochondrial damage amplify innate immune responses by activating their corresponding inflammasome.

#### Mitochondrial-generated double-stranded RNAs activate RLR-MAVS-mediated immunity

**Figure 5.**
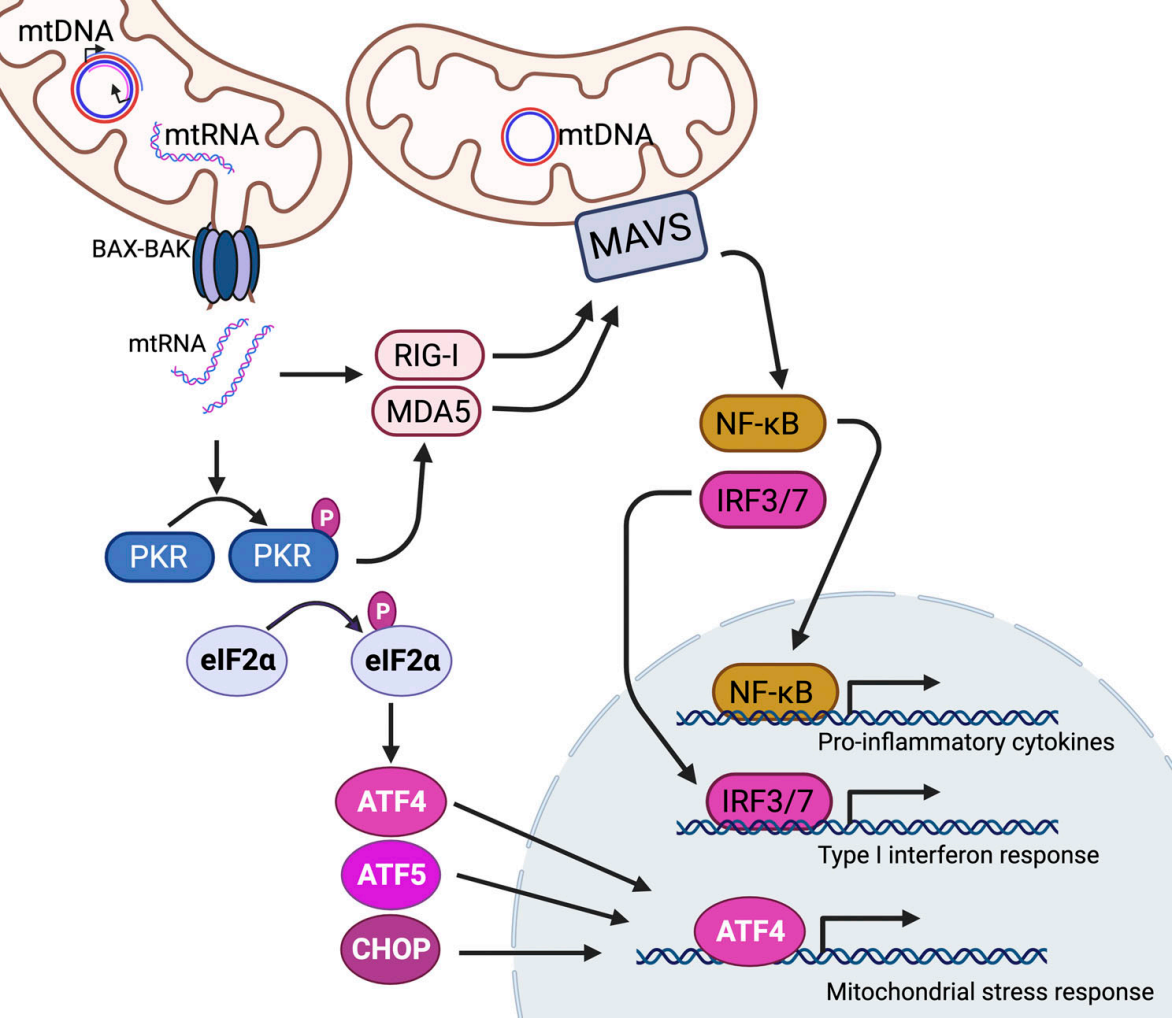
**Cytosolic accumulation of double-stranded RNAs generated during transcription of mtDNAs activates PKR and MAVS.** Cytosolic mtRNA activates RIG-I/MDA5 mediated innate immune response as well as ISR in a manner requiring the kinase PKR. Bidirectional transcription of mtDNAs generates double-stranded mtRNAs, which can escape mitochondria via BAX–BAK-mediated pore formation. Cytosolic mtRNAs stimulate innate immunity by activating RIG-I or MDA5. Once activated, RIG-I or MDA5 induces a pro-inflammatory and type I interferon immune response by increasing NF-κB and IRF3/7, respectively, in an MAVS-dependent manner. Independently, cytosolic mtRNAs bind and activate PKR-mediated ISR activation, inducing the ISR by increasing expression of the transcription factors ATF4, ATF5, and CHOP.

When double-stranded mtDNA-derived RNAs accumulate following transcription, they are usually degraded by the exonuclease polynucleotide phosphorylase (PNPase) within mitochondria. Individuals harboring mutations within the gene encoding the PNPase component PNPT1 have a chronic type I interferon response due to the accumulation of mtRNA within mitochondria, which is ultimately released into the cytosol (Dhir et al., 2018). The release of DAMPs from dysfunctional mitochondria may also activate inflammatory response in cells with wild-type PNPase. Upon sensing dsRNAs derived from mtDNA and/or ssRNA in the cytosol, RIG-I-like receptors (RLRs), including RIG-I or MDA5, assemble the mitochondrial antiviral signaling protein (MAVS), which resides in the mitochondrial outer membrane and functions as a hub for antiviral innate immunity (Seth et al., 2005; Pichlmair et al., 2006). Activated MAVS induces an antiviral response that requires NF-κB and expression of IRF3 and IRF7 (Dhir et al., 2018; Seth et al., 2005; Liu et al., 2015) (Fig. 5).

Interestingly, mtDNA double-stranded breaks caused by mtDNA cleavage at a single site by a TALEN (transcription activator-like effector nuclease) induces the RIG-I-MAVS-mediated innate response in the absence of infection (Tigano et al., 2021). Following mtDNA break or cleavage, BAX/BAK-mediated mitochondrial herniation induces the type I interferon response. Intriguingly, exposure to ionizing radiation also generates double stranded-breaks in mtDNAs, dsRNAs accumulation, and BAX/BAK pore-mediated herniation that induce antiviral type I interferon response (Tigano et al., 2021). In addition to the release of mtDAMPs via pore formation and MDVs, studies also suggest that an increase in mitochondrial membrane potential is required for the mtRNA release and MAVS-mediated antiviral signaling (Koshiba et al., 2011; Yoshizumi et al., 2017; Hooftman et al., 2023). These findings perhaps suggest that the cause or severity of mitochondrial dysfunction plays a role in determining the mechanism(s) by which mtDAMPs reach the cytosol and stimulate the subsequent immune response. Together, mitochondria not only generate double-stranded mtRNA that can initiate innate immune responses but are also where MAVS resides, which amplifies the innate immune response sensed in the cytosol.

Like the MAVS-mediated immune response, protein kinase R (PKR) is also activated by the accumulation of double-stranded RNAs in the cytosol (Kim et al., 2018). During viral infections, PKR-mediated ISR activation impairs viral proliferation via phosphorylation of eIF2α, which reduces the rate of general protein synthesis. In uninfected cells, the majority of the dsRNAs found to interact with PKR are double-stranded RNAs generated during bidirectional transcription within the mitochondria (Kim et al., 2014, 2018; Hirusaki et al., 2017) (Fig. 5). It is known that PKR induces NF-κB activation indirectly by inhibiting IκB, which results in activation of IKK and increased NF-κB-dependent transcription. PKR activation can also directly activate the MAPK pathway amplifying the inflammatory signal intracellularly (Williams, 2001). Activated PKR has also been shown to activate inflammasomes via direct binding (Lu et al., 2012). Thus, PKR activation by mitochondrial DAMPs is an important connection between mitochondrial perturbations and the amplification of innate immune responses.

#### mtDNA induces TLR9-mediated signaling

The Toll-Like Receptor 9 (TLR9) resides on endosomes (Kawai and Akira, 2007) and is activated by binding unmethylated regions of DNA, which can be found in mtDNAs, bacterial genomes, and viral genomes. Similar to the STING-, IRF7-, and NF-κB–mediated pathways, activated TLR9 leads to the induced expression of inflammatory cytokines and type I interferons (Kawai and Akira, 2006). TLR9 activates MyD88, which stimulates NF-κB-dependent transcription of inflammatory cytokines and IRF7-dependent type I interferons (Kawai and Akira, 2006) (Fig. 4). It remains unclear how mtDNAs accumulate within endosomes where theTLR9 binding site is located. However, mtDNAs that escape the autophagy pathway contribute to inflammation (Oka et al., 2012). In addition, intercellular transfer of MDVs may also contribute to the accumulation of mtDNAs within the endosomal system and spread the inflammatory response to neighboring cells (Todkar et al., 2021; Torralba et al., 2016).

Extracellular mtDNAs endocytosed by phagocytic cells are potent stimulators of the immune response (Caielli et al., 2016; Itagaki et al., 2015; Zhang et al., 2010) by activating TLR9 and RAGE (Receptor of Advanced Glycation End Products) (Tian et al., 2007; Julian et al., 2012). In the absence of infection, an increase in circulating cell-free mtDNAs has been demonstrated to contribute to cardiac failure, lupus onset, and chronic stress-associated inflammation (Tripathi et al., 2023; Caielli et al., 2016; Garcia-Romo et al., 2011; Oka et al., 2012). Multiple groups have reported evidence suggesting intercellular transfer of mitochondria and mtDNA (Tan et al., 2015; Dong et al., 2023); however, the mechanisms underlying these phenomena remain to be determined. Potentially, secretion of extracellular vesicles containing mtDNAs, exocytosis, or a lytic type of cell death could result in mtDNA accumulation in the extracellular space, which can activate TLR9 in neighboring cells or tissues. Going forward, it will be interesting to understand the relationship between the release of mtDNAs or double-stranded mtDNAs, which are likely generated in dysfunctional mitochondria, and the activation of the UPRmt or ISR.

#### Apoptosis and mitophagy impair mtDAMP-mediated inflammatory responses

The accumulation of mitochondrial-localized molecules in the cytosol has the potential to elicit robust immune response activation. Multiple programs and activities are in place to limit the uncontrolled release of mitochondrial DAMPs and the activation of inflammatory responses. Apoptosis is a form of cell death that is immunologically silent in that the apoptotic cells do not lyse until they are phagocytosed and degraded within neighboring phagocytic cells. Thus, the mtDAMPs are degraded prior to being exposed to neighboring cells or before entering circulation (Ravichandran, 2011; McArthur et al., 2018).

Apoptosis can be initiated by two different pathways. The extrinsic pathway (or death receptor pathway) is induced by the binding of extracellular ligands to transmembrane death receptors such as TNF (Tumor Necrosis Factor) Receptor 1 (TNFR1) or Fas. The intrinsic pathway (or, mitochondrial pathway) is initiated by BAX and BAK-mediated pore formation on OMM that releases cytochrome c via MOMP (Riley et al., 2018) (Fig. 3). The enlarged pores with hundreds of BAX/BAK complex can release mtDNA via herniation and rupture the inner membrane. Additionally, the assembly of smaller BAX/BAK pores releases cytochrome c and other intermembrane space proteins into the cytosol. Once in the cytosol, cytochrome c promotes the maturation of pro-caspases by assembling with the adaptor molecule apoptosis-protease-activating factor 1 (Apaf-1) to form the apoptosome, resulting in cell death via proteolysis (Kluck et al., 1997; Tait and Green 2010).

Interestingly, prevention of apoptosis increased the inflammatory response in mice (Rongvaux et al., 2014) as apoptotic cells lacking caspases have increased secretion of proinflammatory cytokines due to mtDNA accumulation in the cytosol (Rongvaux et al., 2014). The ineffective clearance of apoptotic cells leads to increased cytosolic mtDNA, active cGAS-STING, and inflammation (White et al., 2014). Furthermore, apoptotic caspases also prevent the overactivation of inflammation by directly cleaving and inactivating cGAS, MAVS, and IRF3 upon viral infection (Ning et al., 2019). Efficient clearance of apoptotic cells by phagocytes further impairs exposure of mitochondrial DAMPs, which could induce subsequent inflammatory cell death and tissue damage (Doran et al., 2020) (Fig. 3).

Consistent with this observation, sublethal cytosolic cytochrome c release via minority MOMP elicits both caspase-dependent (Ichim et al., 2015) and caspase-independent (Kalkavan et al., 2022) DNA damage, contributing to cancer cell survival and tumorigenesis. Interestingly, cytochrome c release from mitochondria that occurs during minority MOMP activates the ISR via DELE1 and HRI (Kalkavan et al., 2022). Rather than causing apoptosis, minority MOMP and ISR activation promote cell survival.

Autophagy is a cell-intrinsic mechanism that also limits uncontrolled immune response activation by eliminating damaged or dysfunctional mitochondria. Autophagy is a cellular process that engulfs cytoplasmic components, including mitochondria, by forming a double-membrane autophagosome that engulfs its cargo and ultimately fuses with a lysosome where the contents are degraded by resident proteases and lipases (Melia et al. 2020). Similar to inhibition of apoptosis, inhibition of autophagosome formation or lysosomal components results in an increase in cGAS, inflammasome, and TLR9-mediated immune responses, triggered by the accumulation of mitochondrial DAMPs in the cytosol (Saitoh et al. 2008, 2009; Yamazaki et al., 2020; Nakahira et al., 2011; Oka et al., 2012).

Mitochondrial autophagy, or mitophagy, is a form of autophagy by which damaged or defective mitochondria are detected by PINK1 and Parkin and targeted to lysosomes for degradation (Killackey et al. 2020). Interestingly, inflammation caused by damaged mitochondria due to mutant mtDNA accumulation was reduced by STING inhibition, suggesting that pathologic hallmarks of Parkinson’s disease, can be mitigated by the inhibition of cGAS-STING (Sliter et al., 2018). Consistent with this finding, mitophagy limits inflammation by degrading damaged mitochondria in different pathologies including sepsis, nephropathy, and cancer (Zhong et al., 2016; Kim et al., 2016b; Chen et al., 2019; Li et al., 2019a, 2019b; Crespo et al., 2020). Alternatively, caspase-1 activation upon inflammasome assembly can impair mitophagy by cleaving Parkin, which augments mitochondrial damage and promotes pyroptosis (Yu et al., 2014). Mitochondrial clearance also occurs via autophagy independent of Parkin. In this case, autophagy is initiated by BAX/BAK pore formation requiring ATG5 and ATG7 to degrade mitochondria and limit inflammation during apoptosis (Lindqvist et al., 2018). Conversely, during oxidative stress, Parkin initiates the recruitment of NEMO (NF-κB essential modulator) to mitochondria, which activates NF-κB signaling and promotes a parallel source of proinflammatory cytokines induced by mitochondria (Harding et al., 2023).

In summary, apoptosis and autophagy are two programs in place to limit unfettered inflammatory response signaling. However, the precise relationship between inflammation and regulated cell death within complex tissues remains to be determined.

#### Retrograde signaling and innate immunity

Prolonged mitochondrial dysfunction can lead to chronic inflammation due to the continuous release of mtDAMPs into the cytosol. Importantly, prolonged innate immune activation by mtDAMPs can cause chronic inflammation which impairs pathogen resistance in the host (DiNardo et al., 2022; de Nooijer et al., 2023; Cohn et al., 2022). Thus, maintenance of mitochondrial integrity by ISR or UPR^mt^ activation antagonizes cytosolic accumulation of mtDAMPs, limiting chronic inflammation (Steinberg et al., 2006; Warren et al., 2023; Kumar et al., 2023). For example, during *P. aeruginosa* infection in *C. elegans*, the UPR^mt^ induces transcription of genes to promote mitochondria function, as well as antimicrobial peptides, both of which are required for survival (Pellegrino et al., 2014; Chen et al., 2016; Kumar et al., 2022). Similarly in mice, ATF5-dependent UPR^mt^ activation promotes survival and maintenance of the enteric barrier which coincides with impaired pathogen spreading during *Salmonella enterica* infection (Chamseddine et al., 2022). In both studies, UPR^mt^ activation improves resistance to pathogens leading to disease tolerance, while limiting perpetual inflammation caused by mtDAMPs. However, some pathogens evolved to escape the host protective mechanisms by suppressing UPRmt. For example, during chronic infection with *P. aeruginosa* in *C. elegans*, the bacterial enzyme acyl-CoA dehydrogenase (FadE2) represses the UPRmt, resulting in host susceptibility (Mahmud et al., 2020).

In addition to improving host resistance, activation of retrograde signaling can mitigate the effects of mtDNA instability. A study demonstrated that mtDNA breaks caused by the targeting of a restriction enzyme to the mitochondrial matrix activate the ISR via DELE1-induced activation of HRI, which recovers mtDNA copy number (Fu et al., 2023). In addition to known ISR-regulated genes, genes associated with inflammation were also observed. However, the mechanism by which DELE1 or HRI activation interacts with inflammatory pathways induced by mtDNA instability remains unclear. Regardless, several studies have demonstrated that ISR activation can downregulate proinflammatory responses. For example, the translation of ATF4 promotes the expression of NRF2, which limits inflammation (Kreß et al., 2023). Furthermore, ISR activation in cancer cells induces expression of the immune suppressive checkpoint inhibitor PD-L1, which impairs T-cell activation to avoid anti-tumoral responses (Xu et al., 2019; Suresh et al., 2020). Thus, the activation of retrograde signaling pathways such as the ISR and UPRmt promotes the recovery of mitochondrial function and integrity, which promotes clearance of cytosolic mtDAMPs and attenuates innate immune response signaling.

#### Concluding remarks

Findings over the last ∼15 years have demonstrated that in addition to roles in energy production, mitochondria function as hubs in diverse signal transduction pathways. Here, we have focused on the role of mitochondrial-nuclear communication in the regulation of mitochondrial proteostasis and the defense against both bacterial and viral pathogens. These signaling pathways allow cells to adapt to both internal and external stimuli emphasizing the integration of the former prokaryote as an essential component of eukaryotic and metazoan life.

While appropriately regulated mitochondria-to-nuclear signaling pathways are protective, dysregulation of these pathways may perturb mitochondrial function culminating in tissue damage, aberrant development, or cell death. Further delineation of the mechanisms by which mitochondria-nuclear communication is regulated will allow for a better understanding of pathological processes and may contribute to the development of therapies for diseases associated with mitochondrial dysfunction and inflammation.
